# Engineering the structural and electrical interplay of nanostructured Au resistive switching networks by controlling the forming process

**DOI:** 10.1038/s41598-023-46990-4

**Published:** 2023-11-12

**Authors:** Giacomo Nadalini, Francesca Borghi, Tereza Košutová, Andrea Falqui, Nicola Ludwig, Paolo Milani

**Affiliations:** 1 CIMaINa and Dipartimento di Fisica, Università degli Studi di Milano, Via Celoria 16, 20133 Milan, Italy; 2 Faculty of Mathematics and Physics, Charles University, V Holešoviˇck ´ ach 2, 18000 Prague 8, Czech Republic

**Keywords:** Nanoscience and technology, Nanoscale materials

## Abstract

Networks of random-assembled gold clusters produced in the gas phase show resistive switching (RS) activity at room temperature and they are suitable for the fabrication of devices for neuromorphic data processing and classification. Fully connected cluster-assembled nanostructured Au films are characterized by a granular structure rich of interfaces, grain boundaries and crystalline defects. Here we report a systematic characterization of the electroforming process of the cluster-assembled films demonstrating how this process affects the interplay between the nano- and mesoscale film structure and the neuromorphic characteristics of the resistive switching activity. The understanding and the control of the influence of the resistive switching forming process on the organization of specific structures at different scales of the cluster-assembled films, provide the possibility to engineer random-assembled neuromorphic architectures for data processing task.

## Introduction

Over the last three decades the advent of the “big data” paradigm in science, business, media, and telecommunications urged the search for solutions of the inability of traditional CMOS-based computing platforms to perform massive real-time data processing at a sustainable hardware and energy cost^1^. The race for extreme miniaturization, beyond the physical limits of CMOS technology, suggested the possibility of developing radically different approaches to computation, based on nanoscale objects such as molecules, nanowires, quantum dots^2–5^. The development of massive fabrication technologies of nanometer-scale logic devices collided with the much higher energetic and manufacturing cost of the top-down lithographic techniques compared to bottom-up approaches and with the nature of nanoscale components usually affected by defects, structural flaws and variability in performances^6,7^. Pursuing the analogy with biological neural networks, the memristor was identified as an artificial alter ego of the neuron and of the synapse thus providing the elemental building block for the fabrication of hardware supporting artificial neural networks at reduced energetic costs^8,9^. The use of memristors, in the mainstream research and technological effort, however is not driving a complete adoption of the mammalian brain architectures^10^, since memristors are organized in deterministic crossbar arrays fabricated with CMOS technology and coupled with traditional microelectronic components^9^, thus making many problems related to CMOS technology still waiting to be solved.

In order to cope with these limitations, several architectures based on defect tolerance, redundancy and reconfigurability were proposed, taking inspiration from the architecture of the mammalian brains^11–14^. Brain activity is based on networks composed by neurons organized according to redundant structure and connected by non-linear synapses, whose organization and activity infer them spatio-temporal correlations^15–18^. The fabrication of interconnected redundant networks based on standard electronic components is beyond the capabilities of top-down technologies^19–21^. Alternative fabrication approaches of hardware based on redundant interconnectivity can rely on random-assembling strategies^11,22,23^. In the last decade, random-assembled networks composed by a large number of non-linear nanoscale junctions between nanoparticles and nanowires have been proposed^2,13,23–25^.

Among random-assembled neuromorphic devices, it has been recently demonstrated that the assembling of gold nanoparticles produced in the gas phase results in nanostructured films exhibiting non-ohmic electric behaviour and complex Resistive Switching (RS) activity^26–29^. These random-assembled systems manifest interesting features as resistive switching events characterized by power law dynamics and long-range interactions^11,26,28^. Resistive switching in Au cluster-assembled devices have been recognized as the result of grain boundaries dynamic reorganization under an applied external voltage^27,28^. However, the phenomena which drive the forming of the reconstructed granular networks and which induce a reconfiguration of the structural and electrical properties of the system from an ohmic to a non-linear electrical behaviour have not been identified yet. Furthermore, the interplay between the morphology and the structure of the activated random-assembled network, from the nano to the mesoscale, and the non-linear electrical behaviour has never been explored systematically.

Here we report the characterization of the influence of the electroforming process on the resistive switching activity with time correlations of cluster-assembled Au nanostructured films (ns-Au). The control of the forming processes acting on the nanoscale and mesoscale re-organization of the nanostructured films allows also to gain a deeper understanding of the interplay between structural and functional electric properties of these systems.

## Experimental methods

### Device fabrication

**Figure 1:**
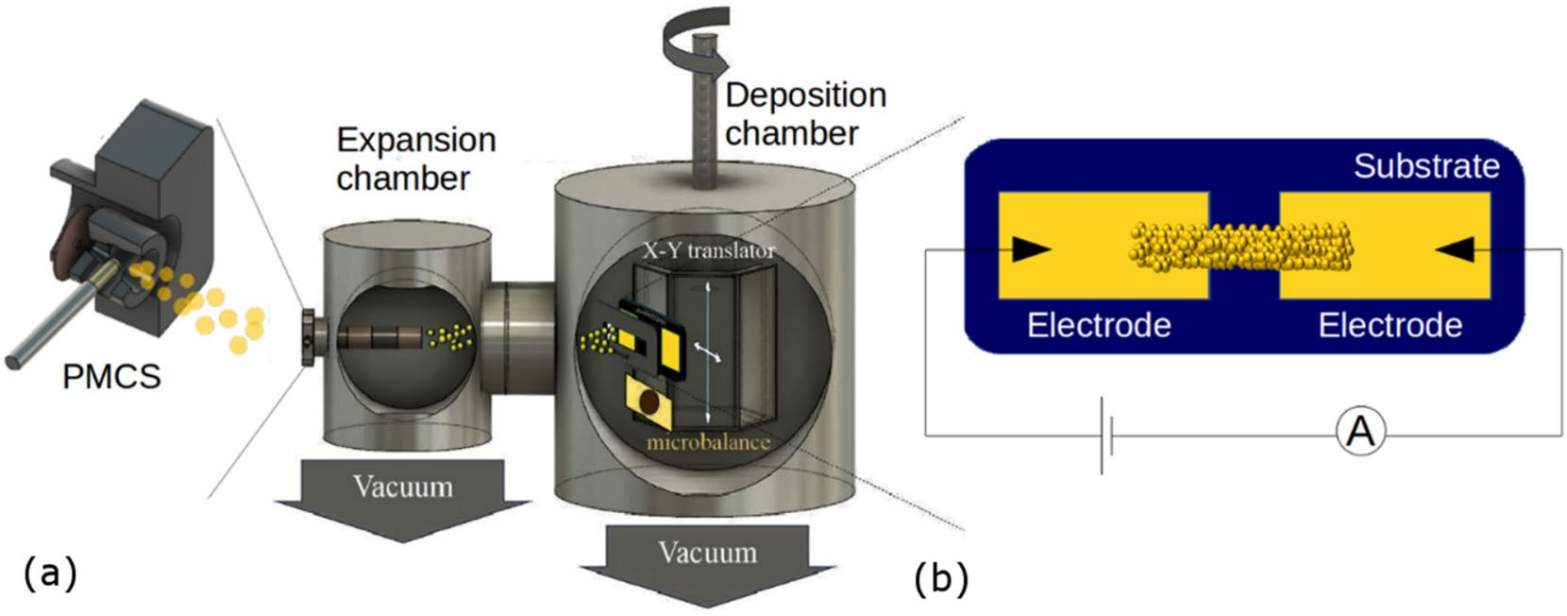
(**a**) Schematic representation (not to scale) of the apparatus for the deposition of cluster-assembled Au films. It consists of a pulsed micro-plasma cluster source (PMCS) mounted on the axis of differentially pumped vacuum chambers; the PMCS produces a supersonic expansion of an inert gas seeded with metallic clusters to form a cluster beam. The beam is intercepted by a substrate placed on a mobile holder (manipulator) in the deposition chamber. Substrates with gold electrodes, previously deposited by thermal evaporation, are mounted on the sample holder and intercept the cluster beam passing through a mask allowing the deposition of stripe of gold cluster-assembled film connecting each pair of electrodes, as it is schematically shown in (**b**). A quartz microbalance attached to the manipulator is periodically exposed to the cluster beam to monitor the amount of deposited material. In situ electrical characterization during the cluster-assembled film growth is performed.

Two-electrode devices based on nanostructured Au films were fabricated using a Supersonic Cluster Beam Deposition (SCBD) apparatus^30^ equipped with a Pulsed Micro Plasma Cluster Source (PMCS) for the production of clusters in the gas phase, as described in detail in Refs.^30–32^. The SCBD set-up (Fig. 1) schematically consists of two differential pumped vacuum chambers. The first chamber is coupled with a PMCS where the production of clusters in the gaseous phase is realized through the ablation of a metal target by a discharge plasma after the injection of an inert gas (Ar). The cluster-inert gas mixture is then extracted to form a supersonic seeded beam that impinges on a substrate fixed on a sample holder perpendicular to the beam trajectory^30^ in the second chamber (Fig. 1a). Two rectangular gold electrodes separated by a gap of 1 mm were deposited by thermal evaporation on the substrate. Figure 1b shows the scheme of a two-electrode device, each of them with typical dimensions of 3 mm × 5 mm. The evolution of the electrical resistance of the cluster-assembled film during the deposition was monitored in situ^26^. This, together with a quartz microbalance, that periodically monitors the amount of deposited material, allows the precise recording of the evolution of the film resistance with thickness^26^.

We used two different substrates: glass and thermally oxidized silicon (SiO_x_). This choice is motivated by the different electroforming processes promoted by the substrate itself, as it will be discussed later on. The lateral dimensions of both substrates are 2 cm × 2 cm. Glass substrates were cleaned by 10 min of sonication in an ethanol bath. Oxidized silicon substrates were produced from silicon wafers through wet thermal annealing^26^ according to a procedure to obtain an oxide layer of about 180 nm. In particular, the oxidation was obtained by a 2-h ramp from RT to 975 °C under a controlled N_2_ atmosphere, followed by 4-h at 975 °C in humid air and finally the cooling to ambient temperature, carried out in N_2_ controlled atmosphere. The silicon substrates were previously cleaned to remove organic contaminants by 10 min bath at 80 °C in a solution made of 5 parts of deionized water, 1 part of NH_3_ part (ammonia water 29%) and 1 part of H_2_O_2_, subsequently ionic contaminants were removed through 10 min bath at 80 °C in 6 parts of deionized water, 1 part of HCl and 1 part of H_2_O_2_.

We deposited fully connected Au cluster-assembled films beyond the electrical percolation threshold, with thickness ranging between 15 and 40 nm, corresponding to as-deposited resistance values in the range 50 Ω < R < 200 Ω. For each type of substrate, 15 samples were produced in different depositions, named from here as ns-Au/glass and ns-Au/SiO_x_. We refer to the samples that underwent to the electroforming process as switched samples.

We studied also an ns-Au film deposited on SiO_x_ ultra flat sample, produced by an oxidation procedure of the SiO_x_ sample by wet thermal oxidation in a quartz tube furnace at 975 °C for 2.5 h in oxygen/hydrogen atmosphere, followed by 20 min in nitrogen to reduce surface defects.

### Electrical characterization

Electrical characterizations were performed using a Keysight E5270 electrometer, both as a voltage source and for the current measurements, remotely controlled via home-made LabView programs to synchronize the application of the voltage and the measurement of the current at the desired sampling rate. The resistance R of the devices was obtained through the Ohm’s law R = V/I by applying a voltage V and measuring the current I in the device. In the case of current measurements at constant applied voltage, the current was sampled every 50 ms for several minutes (20 000 points, equivalent to 1000 s of total application time).

#### Data analysis method

The RS data analysis method is extensively reported in Ref.^28^ and it was performed through a software developed in MATLAB environment, aimed at identifying the electrical resistance temporal evolution under an external voltage applied. Briefly, we performed a threshold analysis to distinguish resistive switching events from random noise. A threshold was set and compared to the relative difference of consecutive resistance values in the temporal resistance series to discriminate from noise. As a result, a switching event is recorded if two consecutive resistance values differ by a number greater than 4 times the standard deviation of the resistance values computed in an interval without switching events, normalized by the average of the selected values^28^. The analysis was carried out for each resistance series measured at different voltage values (± 5, ± 15, ± 25 V). We computed the Inter-Switch-Interval (ISI) distribution by identifying the temporal distance t_ei_ = t_i + 1_ − t_i_ (the inter-event time) of consecutive switches for the entire interval in order to spot the presence of correlations of the switching events in the resistance time series^24,33^. The data are represented in form of probability density distributions. To fit the function that describes the distribution we used the least-squares method implemented in MATLAB environment. We fitted both power law and exponential function and choose the best one comparing the R^2^ coefficients, thus discriminating correlated from uncorrelated resistance time series, respectively represented by the mentioned probability density functions. An ISI distribution which develops according to power laws indicates temporal correlations, while uncorrelated time series are represented by a uniform distribution of switching events, namely the ISI is described by an exponential trend^24,33,34^.

In order to confirm the ISI evaluation and the presence of temporal correlations in sequences of discrete switching events, the analysis proposed by Karsai et al. in^35^ was performed. This consists in counting the switching events which fall into a burst period t _ie_ < Δ t, where Δt is a fixed time interval, longer than the inverse of the sampling frequency. Then the presence of temporal correlations is spotted by comparing the distributions of the number of events that belong to the same burst period, for both the original and shuffled data series in which the latter should show a non-correlated behaviour due to the uniformly distributed shuffled switching events. Uncorrelated independent events in the shuffled data series are therefore represented by an exponential decay in the distribution data tail; conversely, a heavy tail described by a power law distribution indicates the presence of temporal correlation in the data^33,35^.

### Morphological and structural characterization

In order to describe the network morphology at different scales, the nanostructured Au films were characterized by Scanning Electron Microscopy (SEM) imaging, using a Zeiss Sigma microscope, equipped with a Schottky electron source and operating at an acceleration voltage of 5 kV. Several images have been acquired at magnification ranging from 70 to 150 kX. The image processing consisted in binarizing the native SEM images and then in analyzing the geometrical properties of the identified nanostructures. The binarization of the SEM images has been performed by a thresholding strategy based on Otsu’s method, which works well with high-contrast images^36^. All the grayscale values below the threshold level were replaced with 0 (the substrate), while the other ones with 1 (the metal network). Once the binarization step was carried out, the analysis performed through a homemade software that exploits MATLAB functions aims in quantifying the geometrical properties of the network structures, among which:

- The coverage, i.e. the ratio between the projected area occupied by clusters-assembled thin films on the surface and the SEM image scanned area;
- Density of isolated objects (${\#}_{\mathrm{objects}}$/µ${\mathrm{m}}^{2}$) and holes (${\#}_{\mathrm{holes}}$/µ${\mathrm{m}}^{2}$) in the images;
- Density of endpoints (${\#}_{\mathrm{endpoints}}$/µ${\mathrm{m}}^{2}$), i.e. of the endpoints of the skeleton, which is the reduction of all objects in the binary image to 1-pixel wide curved lines. This process, called skeletonization, extracts the center-line while preserving the topology and Euler number of the objects.
- Density of nodes (${\#}_{nodes}$/µ${\mathrm{m}}^{2}$), i.e. of the nodes calculated on the skeleton image.

**Figure 2:**
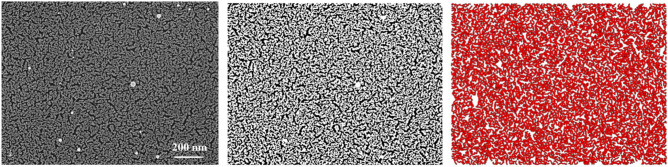
SEM image of ns-Au thin film switched on oxidized silicon substrates (left panel). The corresponding binarized image (middle panel) and the skeleton of the largest object (right panel) are shown.

By using ImageJ software, the width distribution of the smallest nodes connecting different regions of the cluster-assembled network has also been evaluated. In left panel of Fig. 2 the original SEM image of switched ns-Au gold on oxidized silicon is shown. In middle and right panel of Fig. 2 the corresponding binarized image and the skeleton of the largest binarized object are shown, respectively.

The film morphology characterization was also carried out in air using a Multimode AFM equipped with a Nanoscope IV controller (Veeco Instruments). The AFM was operated in Tapping Mode using single crystal silicon tips with nominal radius of curvature of 5–10 nm and cantilever resonance frequency in the range 200–300 kHz. Scan areas were typically 2 μm × 1 μm with scan rates of 1–2 Hz. Sampling resolution was typically 1 nm/pixel.

The microstructure of the thin films was characterized by the XRD measurements performed by the Rigaku SmartLab diffractometer equipped with a Cu rotating anode (wavelength λ = 1.5418 Å). The ns-Au samples with a 5-mm gap between the electrodes were measured in a parallel beam geometry with an incident angle of 0.6°and incident length limiting slit of 5 mm. The electrodes were covered with Si wafers so as not to contribute to the diffraction pattern. To obtain microstructural parameters, the powder diffraction patterns were fitted by a Rietveld method program MStruct^37^.

High resolution transmission electron microscopy (HRTEM) was performed by a Thermo Fischer Themis microscope, equipped with an ultra-bright Schottky electron source and a double spherical aberration corrector. The microscope operated at 300 kV of acceleration voltage and the images were collected by a CMOS 4kX4k Ceta Camera. In view of the HRTEM imaging, the gold film was deposited by SCBD on an amorphous Si_3_N_4_ thin membrane.

### Thermal characterization

The joule effect due to current flow at the different tested voltages during the resistive switching forming process has been monitored by a thermal camera (AVIO R500EX-pro, uncooled micro-bolometric detector, 640 × 480 pixels) with thermal resolution at the thermal scale used (0–500 °C) of 0.5 K and able to achieve a spatial resolution of 52 µm thanks to a germanium macro lens. For each sample a sequence of thermal images has been recorded with a frame rate between 5 and 30 fps, then from the sequence the few frames where temperature underwent a sudden increase/decrease have been extracted. Despite the issue of obtaining reliable temperature from black-body radiation measurement due to the very low value of the spectral emissivity of gold, no significant difference has been detected between the substrate surface and the gold one apparent temperature indicating the gold film emissivity may be higher than the bulk value (0.02)^38^. This can be attributed to the size of gold film (few tens of nm) with respect to the wavelength of the infrared radiation (⁓10 µm). As fully explained in Ref.^39^ the emissivity of the metallic nano-films increases when the thickness becomes comparable to the infrared skin depth, evaluated for Au at ∼10 nm. Considering the Au thin film coverage of about 50–60% on both substrates, to obtain the temperature values of the different IR-images regions, the substrates emissivity was set to 0.9 meanwhile the ns-Au one at 0.5 which is considered the theoretical limit for such metallic thin films^39,40^. Given this uncertainty on the Au thin film emissivity exact value, the present work attention is not focused on the exact absolute temperature reached by the ns-Au thin film but on the relative temperature differences of the identically deposited ns-Au on different substrates during the electroforming process.

## Results and discussion

### As-deposited morphology and resistive switching forming process

**Figure 3:**
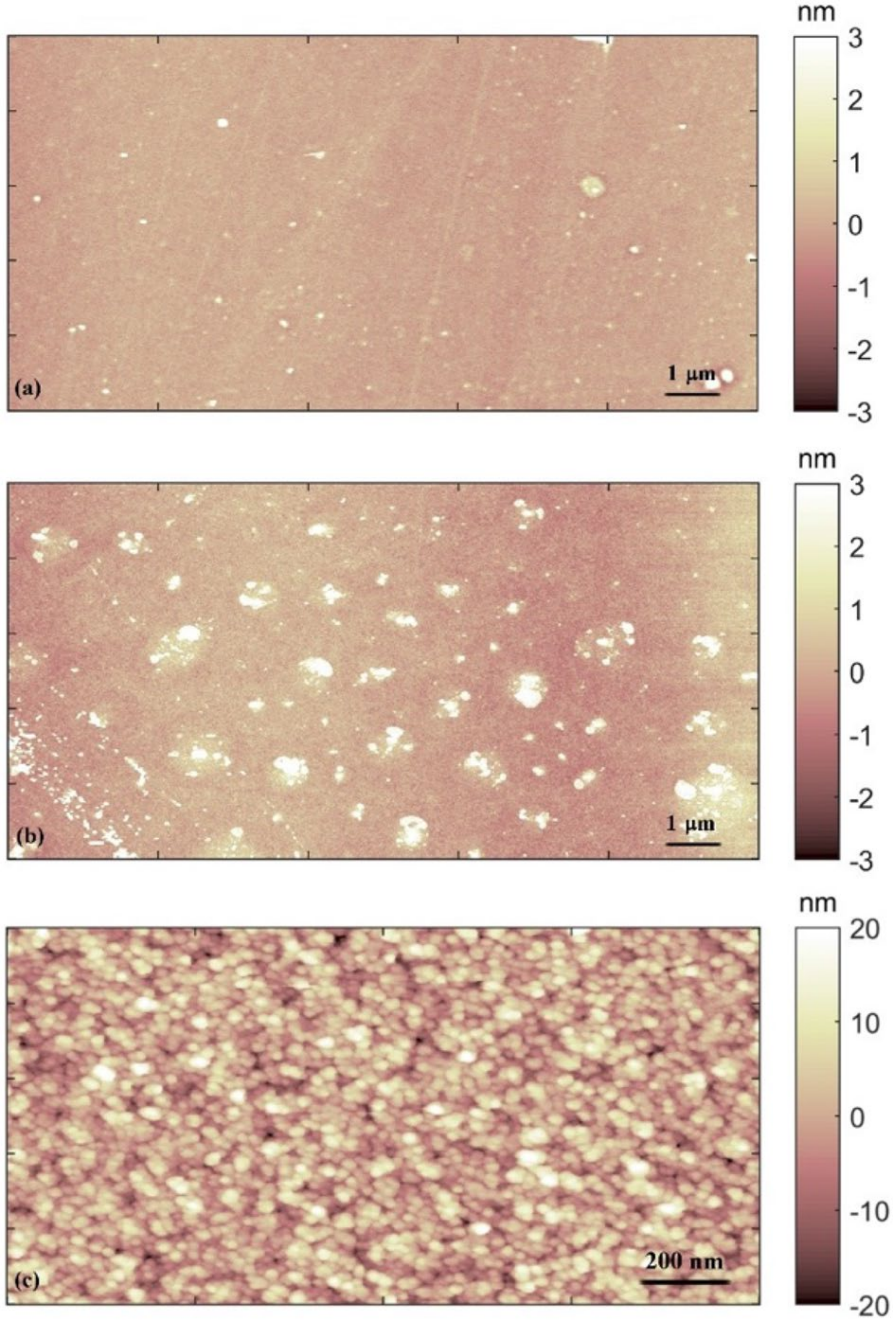
AFM images of the (**a**) SiO_x_ substrate, (**b**) the glass and (**c**) the as-deposited ns-Au thin film on a glass substrate.

The morphology of the ns-Au thin films can be affected by the nanoscale features characterizing the interface of the substrates, i.e. oxidized silicon (SiO_x_) and glass. To test this hypothesis, we evaluated the root-mean-square roughness (Rq) of the substrates, calculated by AFM images as the ones reported in Fig. 3a,b; Rq results 0.34 ± 0.05 nm for SiO_x_ and 0.88 ± 0.3 nm for glass. The morphology of the ns-Au film, a typical AFM image of the as-deposited cluster-assembled gold film is shown in Fig. 3c, appears as a granular and porous matrix composed by a huge number of few nanometres large clusters^26,41^, impinging on the substrate with kinetic energy low enough to prevent them from a post-deposition fragmentation upon the surface^41^. The roughness of the deposited ns-Au film, which increases with the thickness (t) of the film according to a power law (Rq ⁓ t^β^)^41^, is 3.5 ± 0.5 nm. The as-deposited ns-Au films appear with the same granularity and result in same roughness values on both SiOx and glass substrates.

As-deposited gold thin films in the thickness range of 15–40 nm showed ohmic electrical behaviour, as for each explored thickness above the percolation threshold^26^.

The resistive switching forming procedure consisted in circulating high current densities up to 10^9–10^ A/m^2^, for about 90 ± 11 s for ns-Au/SiO_x_ and 30 ± 3 s for ns-Au/glass; this caused the modification of the film morphology, structure, and electrical behaviour^26,42,43^. By high current circulating through the film, we observed a first resistance decrease for granular network reorganization (with a reduction of up the 50% of the initial value), in contrast to the expected ordinary joule-effect related resistance increase observed in metals, followed by a gradual increase of the resistance (up to the double of the initial resistance value in about 1 s) with a final abrupt resistance increase of about 1–3 orders of magnitude^27^. At this point the cluster-assembled films started to exhibit a stable resistive switching activity and we observed non-linear electrical behaviour^26,28,29^. The described RS forming procedure resulted in different electrical and structural evolutions depending on the type of substrates, as reported here after. The ns-Au deposited on ultra flat SiO_x_ and subjected to RS forming procedure remains ohmic even after circulating current densities up to 10^10–11^ A/m^2^.

### Reorganization of the network structure

Mutually correlated interfacial phenomena, as electro-migration and joule heating, occurring at the interface with the ns-Au film can lead the metallic network to a reorganization of its structure and morphology^42,43^. In Table 1 the structural parameters extracted from XRD patterns are reported for the as deposited ns-Au sample, the switched ones on glass and on SiOx and for the ns-Au/SiO_x_ ultra flat sample which manifested ohmic electrical behaviour even after the procedure to trigger the RS activity. It is important to note here that all the XRD measurements were done in ambient conditions and not in-situ during the electrical treatment. The error bars denote the uncertainty of the analysis done by the whole powder pattern fitting program MStruct on measured data.

**Table 1:** Structural parameters calculated from XRD measurements for the as-deposited ns-Au, the switched ns-Au/SiO_x_ and switched ns-Au/glass sample and for the ns-Au/SiO_x_ ultra flat sample after unsuccessful application of the forming process.

Samples	Mean size of crystallites (nm)	Microstrain (%)	Stacking faults (%)
As deposited ns-Au	5.1 ± 0.8	0.7 ± 0.2	13.4 ± 2
Ns-Au switched on SiO_x_	10.5 ± 0.5	0	5.8 ± 0.7
Ns-Au switched on glass	11.7 ± 1.4	0.4 ± 0.2	6.7 ± 1
Ns-Au formed on ultra flat SiO_x_	9.8 ± 0.5	0	5.2 ± 0.6

The RS forming procedure causes an increase in the mean size of crystallites and a decrease in the number of stacking faults, comparable for the systems on both substrates. In the case of ns-Au/SiO_x_ samples after the forming procedure the microstrain, i.e., the distribution of interplanar spacings, vanishes completely pointing to more ordered crystal structure compared to the system on glass substrate. Conversely the persistence of microstrains in the ns-Au/glass system suggests a still granular and rough morphology characterizing the network at the nanoscale. In fact, the surface is known to induce microstrain in the nanostructure and so smaller structures result in enhanced microstrain^44–46^.

**Figure 4:**
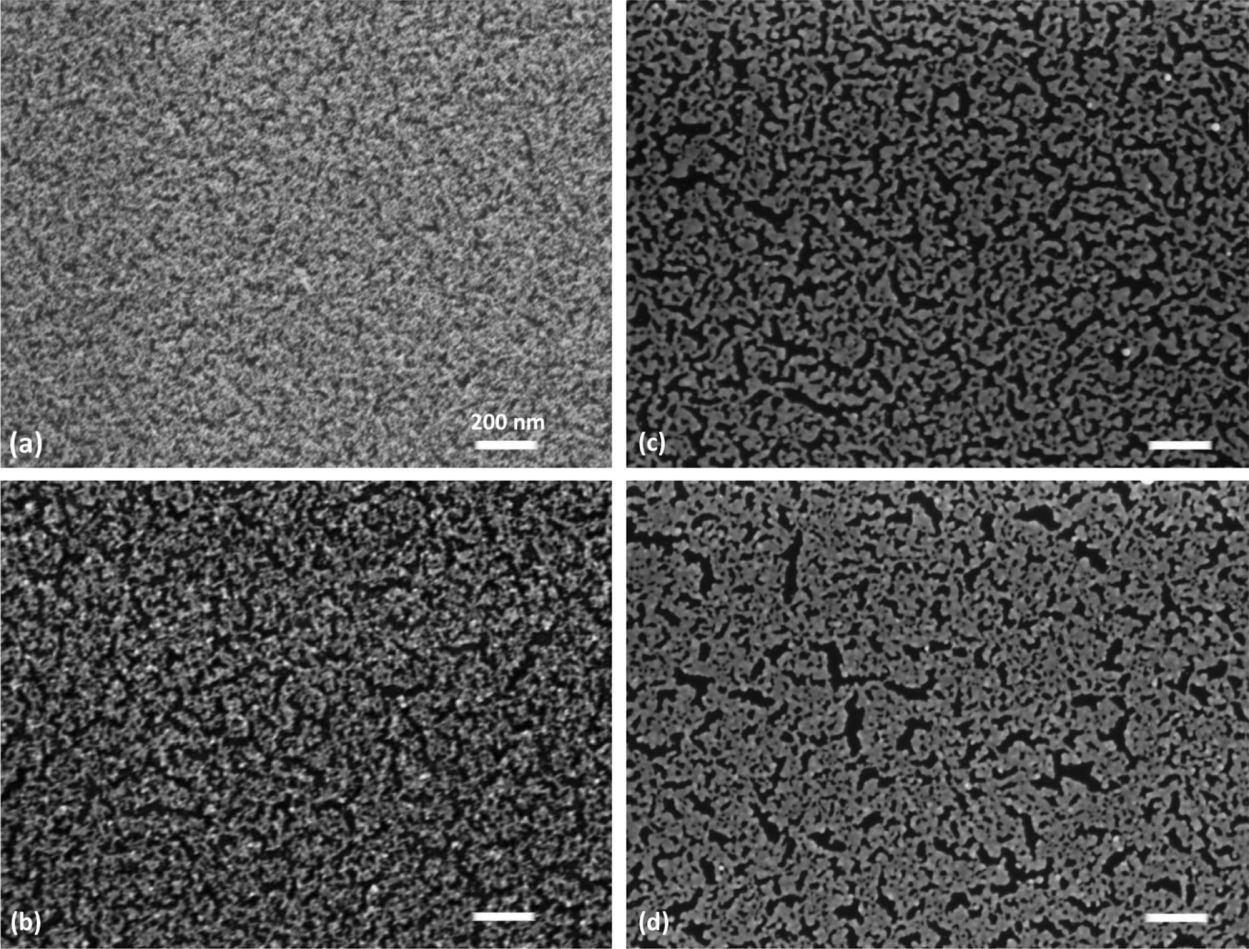
(**a**) SEM images of the ns-Au as-deposited on the glass substrate, (**b**) on the same substrate after the forming process, (**c**) ns-Au/SiO_x_ substrate after the forming process and (**d**) SEM images of the ns-Au deposited on the ultra flat SiO_x_ substrate after unsuccessfully RS forming process, in particular after the application of 35 V.

Figure 4a–d reports the SEM images of the as-deposited ns-Au sample (we reported the one deposited on glass, which is similar to the one deposited on SiO_x_), the two formed ones and the ns-Au unsuccessfully formed on ultra flat SiO_x_; the image of the formed ns-Au/glass sample confirms qualitatively the hypothesis evidenced by XRD measurements. The formed networks are characterized by a complex and highly interconnected network at the mesoscale, while the paths of gold on ns-Au/SiO_x_ sample are more flattened and looks less granular at the nanoscale compared to the ns-Au/glass system.

In order to evaluate the differences in the gold networks formed on glass and on SiO_x_ substrate at the mesoscale, we have performed an analysis of the SEM images by home-made MATLAB routines, described in Materials and Method section, to evaluate the connectivity of the complex structure, on more than five images for each sample. The morphologies of the two formed and switching networks have almost comparable surface coverage (54 ± 1% on SiO_x_ and 61 ± 5% on glass), but they are composed by different density of holes (191 ± 17 #/µ${\mathrm{m}}^{2}$ on SiO_x_ and 236 ± 49 #/µ${\mathrm{m}}^{2}$ on glass), of endpoints (142 ± 20 #/µ${\mathrm{m}}^{2}$ on SiO_x_ and 300 ± 84 #/µ${\mathrm{m}}^{2}$ on glass) and of nodes (854 ± 71 #/µ${\mathrm{m}}^{2}$ on SiO_x_ and 1222 ± 171 #/µ${\mathrm{m}}^{2}$ on glass). In general, the morphology at the mesoscale of ns-Au formed on SiO_x_ is more compact and well connected, compared to the ns-Au/glass system which appears more fragile and further from the continuous layer. This nicely corresponds to the findings from XRD analysis, by which we observed in general more ordered crystal structure for the ns-Au system on SiO_x_ substrate.

Interestingly, the network of the ns-Au/SiOx ultra flat at the mesoscale is more compact (Fig. 4d), since the density of endpoints (75 ± 10), nodes (395 ± 30) and holes (145 ± 35) decreases compared to the ns-Au morphology on glass and on SiO_x_. The coverage of ns-Au switched on SiO_x_ ultra flat is ⁓ 55 ± 3%. It is worth reporting that just minor microstructural differences calculated from XRD measurements were observed between samples deposited on the two types of SiO_x_ substrates, as shown in Table 1. The minor decrease of the overall stacking faults density indicated slightly better ordered crystal structure which again corresponds to more compact structure.

**Figure 5:**
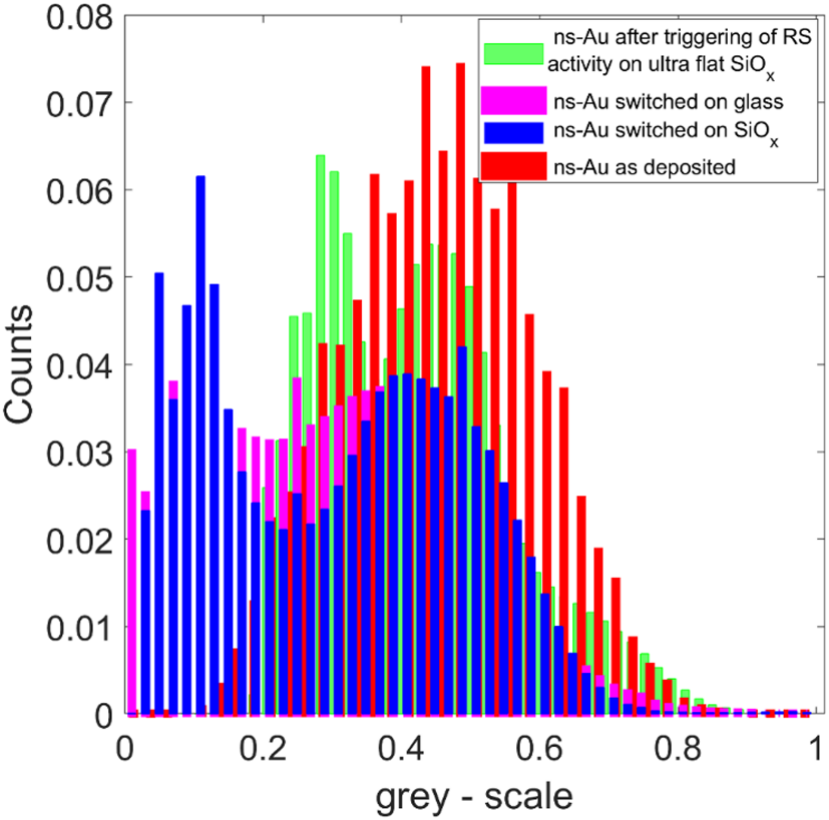
Grey-scale distributions of the SEM images shown in Fig. 4.

The distribution of surface heights in greyscale of the four SEM images (Fig. 5) confirms the layered morphology of ns-Au/SiO_x_ samples at the nanoscale, while more broaden distributions characterize the as-deposited and the ns-Au/glass morphology at this scale.

### Electrical characterization

**Figure 6:**
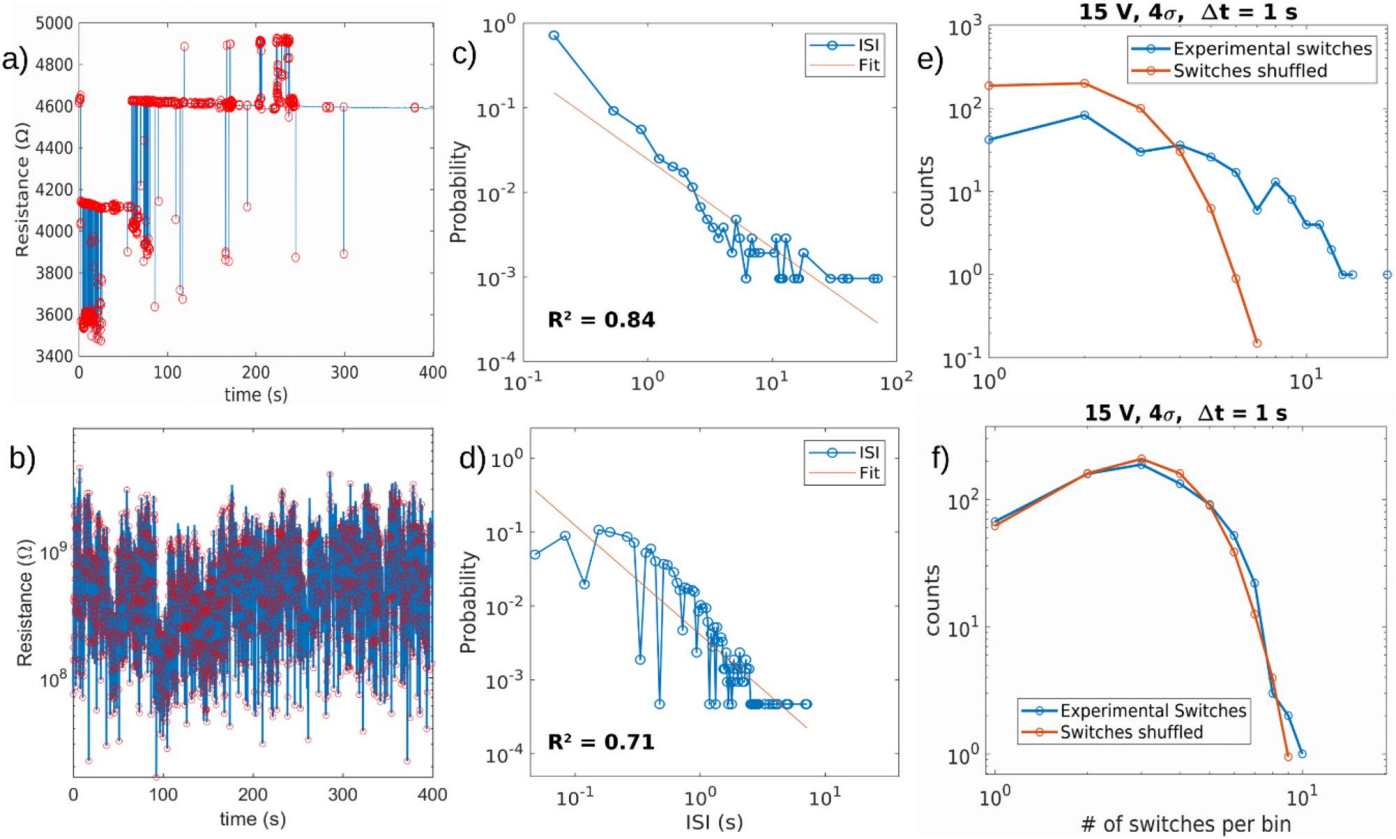
RS activity over a 400 s time window at constant voltages of 15 V for a 23 nm thick ns-Au/SiO_x_ (**a**) and ns-Au/glass (**b**) samples. The switching event (red circle) is identified within the entire set of resistance data (blue data) when two consecutive resistance values differ each other more than 4 times the relative standard deviation of the resistance values computed in a low-noise interval. (**c**,**d**) ISI distributions (blue) in log–log scale fitted with power law line shape (orange) calculated from RS activity on the two samples type, respectively. (**e**,**f**) Distributions of the number of switching events per 1 s intervals of shuffled and unshuffled data series for ns-Au/SiO_x_ and ns-Au/glass samples, respectively.

Non-linear electrical properties of resistive switching Au cluster-assembled devices have been characterized, according to the analysis strategy described in the Materials and Method Section, on three film thicknesses (15 nm, 23 nm, 40 nm). Figure 6a,b reports the resistive switching electrical behaviour manifested by the 23 nm thick Au cluster-assembled films deposited on SiO_x_ and glass substrates respectively. The samples characterized by this thickness has been chosen as representative of all the thicknesses explored, since no difference in morphology as also in the time correlation of the electrical response appears related to a change in thickness. The resistive switching activity of ns-Au/SiO_x_ samples (Fig. 6a) is observed only for applied voltages greater than 5 V, whereas for ns-Au/glass, RS is already observed at voltages larger than 1 V, showing lower stability compared to ns-Au/SiO_x_ for comparable resistance values and so lower current density (this aspect is further investigated in the next paragraph). Furthermore, the activation of resistive switching leads the samples to different resistance values, R_ns-Au/SiOx_ ~ kΩ and R_ns-Au/Glass_ ~ kΩ-GΩ. Moreover, the amplitude of the switching events can be as high as the 100% of the resistance value on glass while on SiO_x_ substrates the switching events are rarely greater than the 10% of the resistance. We have further investigated the discrepancy in the temporal distribution of the switching events in the analysed time window. RS events, marked by red circles in Fig. 6a,b, are grouped for the ns-Au/SiO_x_ samples in burst periods followed by silence periods (no switching activity) of different lengths. On the contrary, the RS events observed for ns-Au/glass samples seem not to be grouped in burst periods but are more uniformly distributed on the time sequence.

In order to quantify the differences of the RS activity time distribution and to evaluate the time correlations of the switching activity, the Inter Switching Interval (ISI)^24,33^ analysis has been performed following the protocol reported in Materials and Methods section. ISI distributions for ns-Au/SiO_x_ and ns-Au/glass samples are respectively shown in Fig. 6c,d in logarithmic scale from which the mentioned differences are quantitatively evaluated. The RS events grouped in small time windows contribute to the left side of the distribution, while the right side of the distributions considers the events separated by longer time periods; the ns-Au/SiO_x_ samples have a distribution tail much more extended than ns-Au/glass. To quantify the time correlation degree systematically, six different ISI distributions were computed for six different voltage values (± 5 V, ± 15 V, ± 25 V) for each sample. Then the obtained distributions were fitted both with exponential and power law line shapes. The power law distribution manifests a heavy tail which indicates the presence of time correlations, on the contrary the exponential distribution represents independent uncorrelated events^47^. Thus, comparing the R^2^ coefficients obtained by the two fitting procedures it was possible to observe a higher correlation degree with power law for ns-Au/SiO_x_ samples than for ns-Au/glass. No significant differences within each of the two systems emerge from the electrical behaviour at different constant voltages, for both the polarities, as well as for different thicknesses. Quantitatively, the subtraction of the power law and exponential R^2^ fitting coefficients is systematically greater for ns-Au/SiO_x_ samples compared to ns-Au/glass ones indicating a better agreement to a power law distribution for the former system, as reported in Table 2.

**Table 2:** Average R^2^ coefficients of the fitting procedures (for all the applied voltage values: ± 5 V, ± 15 V, ± 25 V) for power law and exponential line shapes both for ns-Au/SiO_x_ and ns-Au/glass samples.

	R^2^ power law (ISI)	R^2^ exponential (ISI)	ΔR^2^ (ISI)	Discrepancy
Ns-Au/SiO_x_	0.73 ± 0.07	0.23 ± 0.01	0.5 ± 0.01	0.5 ± 0.05
Ns-Au/glass	0.65 ± 0.01	0.3 ± 0.011	0.35 ± 0.01	0.28 ± 0.03

Difference of the R^2^ coefficients and discrepancies between the shuffled and unshuffled distributions of the number of switching events per 1 s large bin are reported.

We also performed a further analysis following Karsai et al. method^35^, also reported in Materials and Methods section. The distribution of the number of switching events belonging to intervals of 1 s are reported in Fig. 6e,f for the two different substrates, both for shuffled and unshuffled time series. For unshuffled time series, the switching events are uniformly arranged thus the resulting number of events per time interval distribution does not show the tail expected for grouped events as for the case of unshuffled ns-Au/SiO_x_ samples data series which indicates the presence of correlations. On the contrary, for ns-Au/glass samples the distributions of the unshuffled data do not show a significant departure from the shuffled series. To quantify the difference between shuffled and unshuffled distributions the discrepancy was defined as the difference of the normalized distributions, which is also reported in Table 2. The discrepancies computed for all the analysed data series for each sample strengthen the evidence of a higher degree of time correlations for ns-Au/SiO_x_ samples and a lower correlation for ns-Au/glass samples. We argue that the correlation degree discrepancy is due to the morphological and structural differences of the Au cluster-assembled films due to the forming process previously reported and further discussed in the following.

### In situ thermal characterization of the RS forming process

We characterized the evolution of the temperature of the nanostructured films during the resistive switching forming process with a thermo-camera to gain a deeper insight in the mechanisms responsible for the network reorganization. The main electrical and thermal features characterizing the switching triggering of the ns-Au deposited on glass and on SiO_x_, obtained by electrical measurements at constant voltage and by the IR-video acquired by the thermocamera, are reported in Table 3.

**Table 3:** Thermal and electrical quantities (time, temperature, current density, voltage and energy) measured during the forming of the switching activity for ns-Au/SiO_x_ and ns-Au/glass samples.

Substrate	t_sw_ [s]	T_sw_ [°C]	J_sw_ [A/m^2^]	V_sw_ [V]	t_sw_*P_diss_/s_f_ [J/nm]
Ns-Au/SiO_x_	90 ± 11	248 ± 8	(3 ± 0.2) ·10^10^	33 ± 3	40 ± 5
Ns-Au/glass	30 ± 3	430 ± 14	(4 ± 1) ·10^9^	8 ± 1	0.6 ± 0.1

Each value is obtained by averaging over all the tested samples. t_sw_ is defined as the time in which a resistance variation was observed; T_sw_, J_sw_, V_sw_ are the pointwise temperature, current density and voltage at the time of the appearance of the switching, while the last column represents the energy employed during the procedure to form the switching activity normalized by the cluster-assembled film thickness.

**Figure 7:**
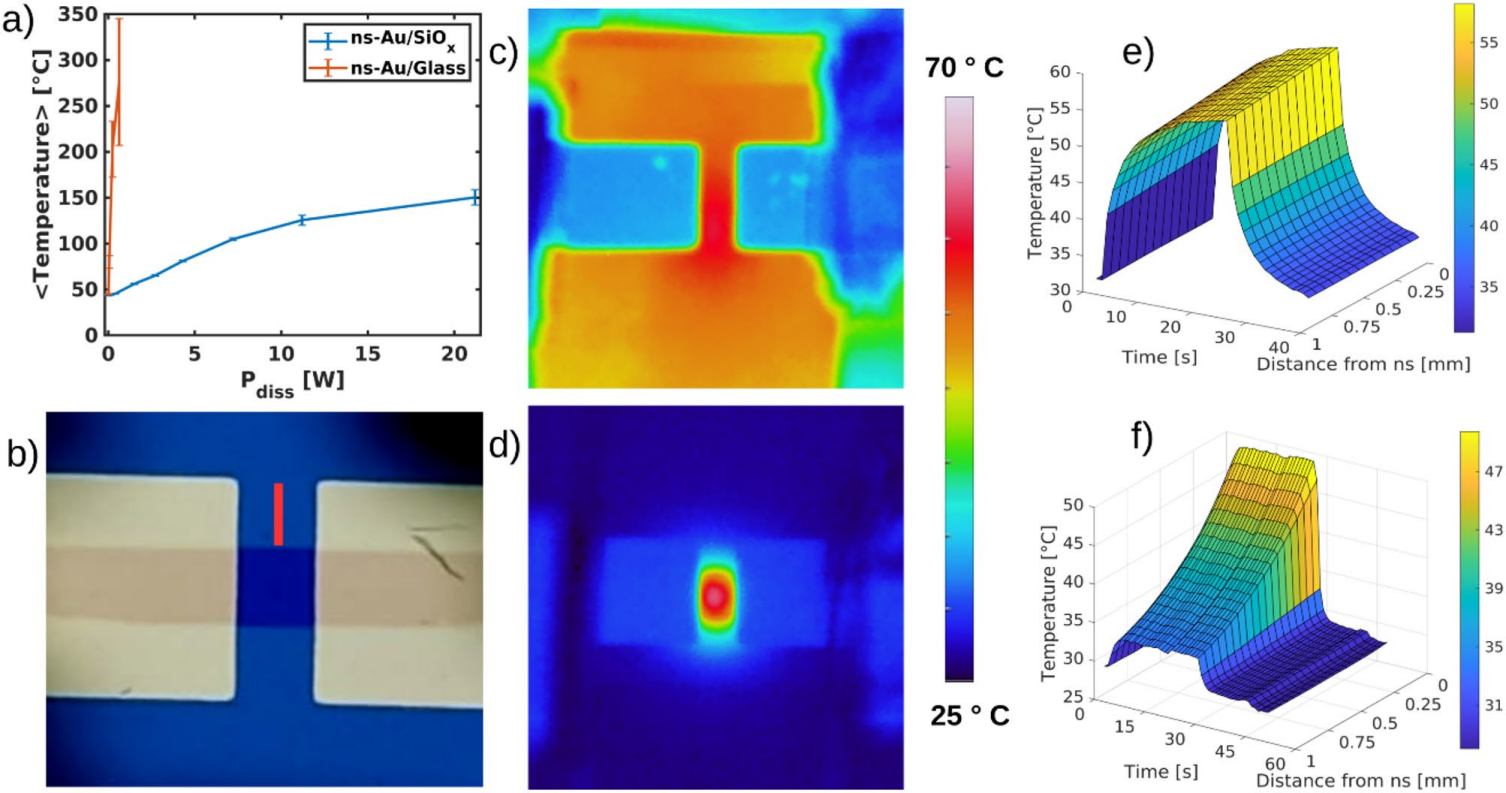
(**a**) Average temperature reached by the ns-Au film area as a function of the electrical dissipated power for glass and SiO_x_ substrates. (**b**) Optical image of SiO_x_ substrate equipped with electrodes bridged by the ns-Au film (shaded stripe in the middle), the red bar (1 mm) represents the area from which figure (**e**) and figure (**f**) were computed. (**c,d**) IR-images of the cluster assembled films respectively deposited on SiO_x_ and glass over which the same temperature range is observed at 25 V and 3 V respectively. Heat dissipation is much more localized in the case of glass. (**e,f**) Temperature of the substrates along the red stripe shown in (b) as a function of the time and distance from the ns-Au film for SiO_x_ and glass, respectively. At time t = 0 s, the power supply is turned on and the sample starts to heat up until the voltage source is turned off and the samples start to cool down. The well localized heat spot is evident in the glass case as like as the faster heating and cooling resulting from different thermal conductivity of the substrates.

The thermo-camera measurements show that SiO_x_ substrates better dissipate the heat than glass, as it was expected. This is clearly demonstrated by the temperature measurements of the nanostructured film area as a function of the dissipated electrical power, computed as P = I · V, where I and V are the applied current and voltage during the switching procedure, respectively, showing that much higher temperatures are reached on the glass substrates even at lower dissipated power (Fig. 7a). Moreover, due to the different thermal diffusion coefficients, the heat dissipated is much more localized close to the nanostructured thin film when considering glass substrates, (see Fig. 7c,d for comparison). Heat localization was quantified by computing the spatial derivatives in a region perpendicular and next to the nanostructured film, as schematically reported in Fig. 7b. Spatial derivatives were evaluated from the graph showed in Fig. 6e,f in which the substrate temperature is showed as a function of time and distance from the nanostructured films. These are proportional to the spatial gradients and in the case of SiO_x_ (see Fig. 7e) they are almost constant, meanwhile the functional shape of the glass spatial derivatives are power laws, which indicates a much stronger heat localization due to lower heat conductivity of glass. Furthermore, time derivatives of the glass substrates have similar functional shape compared to SiO_x_ but they are 2–4 times higher, pointing out that SiO_x_ substrates need more time to heat up and to cool down due to the higher value of its thermal conductivity. Probably, this extra time could also enable the better ordering of crystal structure, which is observed for the SiO_x_ substrate.

The differences of the substrates thermal properties cause the discrepancies observed in the RS forming process suggesting that the onset of the RS activity is due to different relative magnitudes of the thermal phenomena involved. In the glass case the onset of the RS activity was obtained in relatively small times, as reported in Table 3, compared to SiO_x_. Moreover, the temperatures reached on glass substrates were much higher and localized compared to SiO_x_ and the current densities were at least one order of magnitude lower than for SiO_x_. Glass provides the nanostructured film much higher temperatures in less time, modifying the structural properties of the cluster-assembled film mostly as a fast-annealing effect on more localized regions. On the other hand, SiO_x_ dissipates better heat and so higher currents and longer times are needed to trigger the RS activity. In this case, electromigration is probably more relevant, compared to thermic effects due to the lower temperatures reached.

### Discussion on structural and electrical interplay

**Figure 8:**
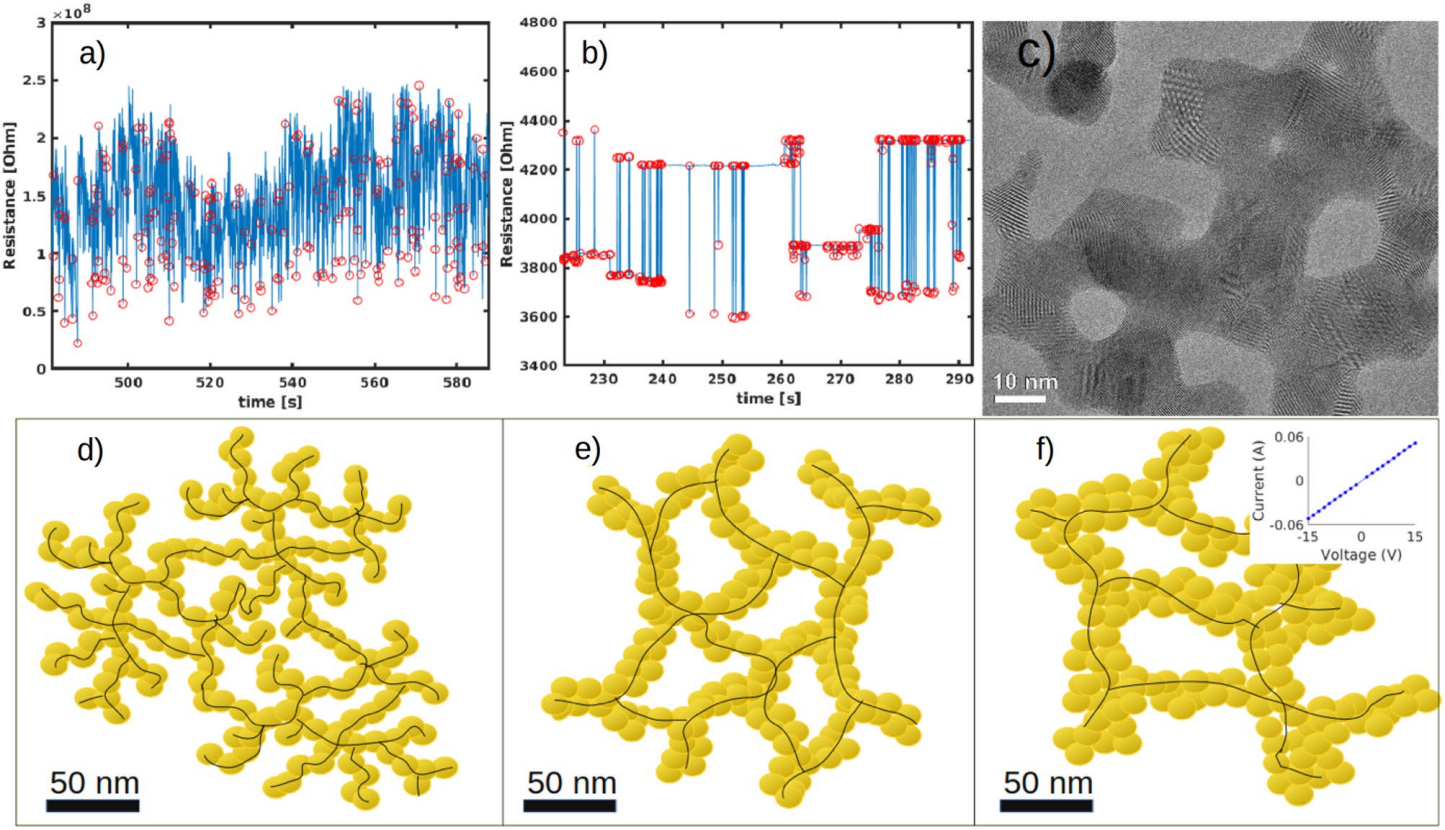
(**a**,**b**) Constant voltage characterization (15 V) of Au cluster-assembled films deposited on glass and SiO_x_ substrates, respectively. The former shows more uniformly distributed RS events, the latter shows multiple metastable resistance levels and burst activity. (**c**) HRTEM image of a typical region of the as-deposited cluster-assembled Au film on Si_3_N_4_ TEM grid. Each white dot is a single atomic column, and parallel dots lines are the lattice planes. The zones where several parallel dots lines, i.e., lattice planes, are observed correspond to crystal domains. The current flow changes grain boundaries orientation giving rise to RS events. (**d**) Schematic representation of ns-Au network evinced by SEM images reported in Fig. 4b: yellow dots represent crystal grains; black lines represent possible current paths. Ns-Au network on glass substrate manifests high density of branchpoints, endpoints and a single hierarchical level defined by the nanoscale features characterized by the typical electrical behaviour (**a**). (**e**) ns-Au network on SiO_x_ substrate is better connected than on glass and manifests two hierarchical levels defined by nanoscale grains and mesoscale reorganization, responsible of the electrical behaviour (**b**). (**f**) Ns-Au network on defect-free surface SiO_x_ substrate present even thicker features preserving ohmic conduction channels. The inset reports the I-V curve of ohmic ns-Au on this flat and low surface defects SiO_x_ substrate.

In order to understand the effect of the resistive switching forming process on the different electrical properties of ns-Au/glass and ns-Au/SiO_x_, reported in Fig. 8a,b, we investigated the corresponding morphological and structural organization of the two networks. It is worth to remember that the resistive switching in Au cluster-assembled films is a consequence of the grain boundaries reorganization due to current-induced local Joule heating, which modifies continuously the nanostructure of the system, as demonstrated in previous works^26–29^.

Figure 8c reports a HRTEM image of ns-Au in which grain boundaries are visible. The modification of the grain boundaries changes the local resistivity of the film and consequently the current redistributes in the neighbour junctions of the metal film^26,28,41^, in a non-trivial recurrent manner. In fact, the redistribution of the current occurs not only as a function of the nanoscale features of clusters, but it also depends on the morphology of the network at the mesoscale, which influences the spatial propagation of the current, redistributed through the ns-Au thin film, and thus the global film resistance dynamic^26^. We took into consideration the mesoscale reorganization of the clusters network to explain the different electrical behaviours reported in section "Electrical characterization", because there were no notable differences in the nanoscale characteristics of the two types of samples, such as grain dimensions and nanoscale defects that emerged from the XRD measurements.

Ns-Au films deposited on SiO_x_ substrates are better connected than on glass, since they manifest bigger connections among islands at the mesoscale. On the other hand, despite the coverage of the two samples is approximately the same, ns-Au/glass samples show more holes, more branchpoints and more ending points, stressing a more fragile and intricated structure composed by thinner and more tangled connections, compared to ns-Au/SiO_x_ samples. Thus, the Joule heating effect due to a further application of constant voltage could be more detrimental for the small connections of Au clusters on glass, leading to severe junction rupture and continuous current redistribution to the neighbours in several pathways, which determines the great continuous resistance changes observed (up to 100% of the film resistance value, Figs. 6b and 8a). Conversely on ns-Au/SiO_x_ samples the lower number of branchpoints and the slightly larger size of the paths promote a correlated current redistribution and probably allow the perturbation propagation toward more distant regions, enhancing the mutual interaction of distinct parts of the system, resulting in the observed correlated resistance changes (up to 10% of the film resistance value, Figs. 6a and 8b).

Moreover, we observed more frequent resistance changes for ns-Au/glass samples compared to ns-Au/SiO_x_ and this could be again a consequence of the less compact and badly connected network at the mesoscale for the former system, schematically shown in Fig. 8d. In fact, smaller morphological features, characterized previously by SEM analysis and shown in Fig. 5, require less current and less time to be modified resulting in a continuous current redistribution due to grain boundary modification resulting in frequent resistance changes. On the contrary, Au clusters films connections on SiO_x_ substrate, schematically reported in Fig. 8e, are more compact and well-organized thus much more current and time are needed to significantly modify the mesoscale junctions leading to longer periods of time in which no resistance changes above the noise level.

The close interplay between structural and electrical properties of cluster-assembled gold films is further confirmed by the study of a ns-Au sample deposited on flat and less defective SiO_x_ substrates. This ns-Au/SiO_x_ ultra flat sample manifested ohmic electrical behaviour even after the procedure to trigger the RS activity because the mesoscale connections are even bigger than in the SiO_x_ case so preserving ohmic conduction channels.

In ns-Au/SiO_x_ samples the nanoscale structure made of grain of 10 nm size and a mesoscale morphology characterized by islands and larger mutual connections than those of the ns-Au/glass samples, define a structural hierarchy which translates in a temporal hierarchy the resistive switching events; namely the time required to modify the nanoscale structural features could be lower compared to the time required to modify the mesoscale structural features, thus justifying the observed power-law distribution of the electrical time series^25^. Conversely, cluster networks on glass manifest less defined hierarchical levels separation, due to the smaller size of the mesoscale features (~ 10 nm), thus resulting in uniformly distributed RS events, namely in uncorrelated electrical activity. Moreover, the ns-Au network on ultra-flat SiO_x_, Figs. 4d and 8f, present even thicker morphological features compared to ns-Au/SiO_x_ network, Figs. 4c and 8e, thus not showing resistance variations and preserving ohmic conduction channel. Interestingly, in literature^48^ there are reported other examples of networks which by presenting more hierarchical levels manifest complex critical behaviour thus being characterized by power laws distributions in their dynamics as like the ones we observed in the temporal distributions of the RS activity. The hierarchical levels of the cluster-assembled film deposited on SiO_x_ organized into greater complexity promotes the critical^48^ and therefore the more correlated behaviour^47^ of the system, compared to the ns-Au/glass sample. This evidence stresses not only that the ns-Au/SiO_x_ devices RS activity exhibits similar statistical properties as the one observed in biological neuronal networks^16–18,49^, but more interestingly that this activity is related to the ns-Au/SiO_x_ network connectivity which is higher than in the case of ns-Au/glass network. The latter is more disordered and with less hierarchical levels separation, morphology that brings it far from the mammalian brain network which manifests in a hierarchical topology^50,51^.

## Conclusions

We have characterized the physical processes underlying the resistive switching forming of cluster-assembled nanostructured Au films: local thermal effects and electromigration induced by the current flowing in the systems cause a structural reorganization at the nano and mesoscale. The thermal dissipation properties of the substrates determine the relative weights of the contribution of these processes and hence the resulting reorganization of the network.

The reorganization of the grain-boundaries during the forming process, considered the physical phenomena at the base of the RS activity in Au cluster-assembled devices^26–29^, is of particular relevance together with the mesoscale formation of bridges between different nanoscale islands. Fine differences in the network reorganization on this scale correspond to specific temporal correlation of resistive switching events; the hierarchical and modular structure of the Au cluster-assembled thin film on each specific scale makes the system complexity great and ordered enough to promote an organized current redistribution, subsequent to RS events due to grain boundary dynamic, resulting in time correlated electrical activity.

Our results stress the role of the morphological complexity in the resulting non-linear electrical activity of Au cluster-assembled based devices; the choice of substrates with different thermal properties and the tuning of electrical parameters during the RS activation open the way to the engineering of the resistive switching dynamics, with defined time correlations and RS amplitude, aiming at the development of bottom-up neuromorphic hardware technologies with suited electrical properties for specific data processing tasks .

## Data Availability

The datasets generated during and/or analysed during the current study are available from the corresponding author on reasonable request.
